# Pressurized intraperitoneal aerosol chemotherapy (PIPAC) in multimodal therapy for patients with oligometastatic peritoneal gastric cancer: a randomized multicenter phase III trial PIPAC VEROne

**DOI:** 10.1515/pp-2022-0111

**Published:** 2022-06-07

**Authors:** Francesco Casella, Maria Bencivenga, Riccardo Rosati, Uberto Romario Fumagalli, Daniele Marrelli, Fabio Pacelli, Antonio Macrì, Annibale Donini, Lorena Torroni, Michele Pavarana, Giovanni De Manzoni

**Affiliations:** Department and of Surgical, General and Upper GI Surgery Division, Odontostomatologic, Maternal and Child Sciences, University of Verona, Verona, Italy; Department of Surgery, San Raffaele Hospital, San Raffaele Vita-salute University, Milan, Italy; Digestive Surgery, European Institute of Oncology, Milan, Italy; Department of Surgery, Siena University Hospital, University of Siena, Siena, Italy; Surgical Unit of Peritoneum and Retroperitoneum Surgery, Fondazione Policlinico Universitario A. Gemelli, IRCCS, Rome, Italy; Peritoneal Surface Malignancy and Soft Tissue Sarcoma Program, University of Messina, Messina, Italy; General and Emergency Surgery, Santa Maria Della Misericordia Hospital, University of Perugia, Perugia, Italy; Department of Diagnostic and Public Health, Unit of Epidemiology and Medical Statistics, University of Verona, Verona, Italy; Department of Oncology, University of Verona, Verona, Italy

**Keywords:** gastric cancer, intraperitoneal chemotherapy, peritoneal metastases, PIPAC, systemic chemotherapy

## Abstract

**Objectives:**

Peritoneal carcinomatosis is the most frequent site of metastases in patients with gastric cancer. Current standard treatment is palliative systemic chemotherapy with very poor prognosis. Cytoreductive surgery (CRS) combined with hyperthermic intraperitoneal chemotherapy (HIPEC) resulted in long-term benefits in selected patients. Among patients with peritoneal carcinomatosis, a distinctive subset is oligometastatic disease which is characterized by low metastatic burden. Pressurized intraperitoneal aerosol chemotherapy (PIPAC) is a recent technique of intraperitoneal chemotherapy used in combination with systemic chemotherapy with promising results.

**Methods:**

PIPAC VER-One is a prospective, randomized, multicenter phase III clinical trial that aims to evaluate the effectiveness of the use of PIPAC in combination with systemic chemotherapy in patients with gastric cancer and synchronous positive peritoneal cytology and/or limited peritoneal metastases (peritoneal cancer index [PCI] ≤6). Patients will be randomized into two arms: arm A (control) treated with standard systemic chemotherapy and arm B (experimental) treated with a bidirectional scheme including PIPAC and systemic chemotherapy.

**Results:**

Primary endpoint is the secondary resectability rate. Secondary endpoints are: overall survival (OS), pregression-free survival (PFS), disease-free survival (DFS), histological response assessed both on primary tumor and peritoneal lesions, quality of life (QoL), complication rate (CTCAE v5), and incremental cost-effectiveness ratios (ICER).

**Conclusions:**

The role of PIPAC in multimodal treatment for oligometastatic gastric cancer will be investigated in this trial.

## Introduction

Gastric cancer (GC) is responsible for over one million new cases in 2020 and an estimated 769,000 deaths, ranking fifth for incidence and fourth for mortality globally [1]. The peritoneum is a common metastatic site in gastric cancer. In a recent population-based study, the incidence of synchronous peritoneal metastases was 21%. This rate rises to 40% if we consider studies that includes laparoscopy in the staging pathway [2].

Despite recent advances in the understanding of disease biology and drug development, the prognosis of gastric cancer patients with synchronous peritoneal metastases treated with systemic chemotherapy only, is still poor [3]. In this scenario, growing evidence has suggested that a subset of patients with limited metastatic spread might achieve long-term disease control from a multimodality treatment strategy. These patients could be defined as “oligometastatic” gastric cancer patients with peritoneal involvement.

To date, very few studies report prolonged survival after surgical resection of oligometastatic gastric cancer patients; moreover, there is little evidence on peritoneal-only disease [4].

The ongoing RENAISSANCE trial, a multicenter randomized clinical trial (RCT), comparing the effect of chemotherapy alone vs. chemotherapy followed by surgery for patients with oligometastatic esophagogastric adenocarcinoma (EGAC) is anticipated to shed light on the best treatment modality [2]. Of note, an aggressive multimodal treatment combining surgery plus systemic chemotherapy could be ineffective in cases oligometastatic GC with peritoneal involvement. Adding an intraabdominal treatment could be the key in such patients.

Pressurized intraperitoneal aerosol chemotherapy (PIPAC), a recent technique of intraperitoneal chemotherapy that can be used in combination with systemic chemotherapy and showed promising results in the palliative setting [5], should be evaluated as a promising tool in the therapeutic algorithm of peritoneal-only oligometastatic GC.

The aim of this study is to evaluate the efficacy of PIPAC in combination with systemic chemotherapy and radical-intent surgery for treatment of peritoneal-only oligometastatic GC.

More in detail, this is a phase III trial comparing two different treatment strategies: the current standard of treatment for gastric cancer with synchronous limited peritoneal metastases (that is systemic chemotherapy with possible radical-intent surgery) will be compared with an experimental treatment that provides in addition to standard systemic chemotherapy, every two cycles of systemic chemotherapy, the administration of PIPAC; moreover patients in the experimental arm will undergo to radical-intent surgery unless progression disease is detected.

## Materials and methods

### Protocol overview

This is a prospective, open label, randomized multicenter phase III clinical study that aims to evaluate the effects of PIPAC combined with systemic chemotherapy vs. intravenous systemic chemotherapy alone on patients with gastric cancer and synchronous positive peritoneal cytology and/or limited peritoneal metastasis (peritoneal cancer index [PCI] ≤6). Patients will be randomly assigned in a 1:1 ratio to arm A: intravenous chemotherapy (FOLFOX) vs. arm B: intravenous chemotherapy (FOLFOX) plus PIPAC with cisplatin and doxorubicin.

### Main inclusion criteria

– Age between 18 and 75 years.
– Primary resectable gastric cancer with positive peritoneal cytology and/or low burden peritoneal metastases (PCI ≤6) confirmed by laparoscopy.
– Signature of written informed consent.
– ECOG PS 0-1.

### Main exclusion criteria

– Extraperitoneal metastases.
– PCI >6.
– Gastro-esophageal junction tumor of esophageal relevance (Siewert I-II).
– Impossibility of local radical resection (duodenal involvement, pancreatic involvement, and infiltration of hepatoduodenal ligament).
– Previous allergic reactions to cisplatin or doxorubicin.
– Hemorrhagic or occlusive manifestation of the primary tumor with palliative surgery needed.
– ASA IV.
– Positivity for Epstein-Barr virus (EBV), microsatellite instability (MSI), and human epidermal growth factor receptor 2, (HER2) on diagnostic biopsies.
– Pregnancy and breastfeeding.
– Contraindication to any drug contained in the chemotherapy regimen.
– Hepatic impairment (aspartate aminotransferase [AST]/alanine aminotransferase [ALT] >3 times normal values, ALT >3 times normal values, bilirubin >1.5 normal values).
– Ischemic/hemorrhagic stroke in the last 6 months.
– Acute myocardial infarction in the last 6 months.
– Moderate/severe heart failure (NYHA III-IV).
– Leukopenia <2,000/μL.
– Thrombocytopenia <100,000/μL.
– Active hepatitis B or C.
– HIV infection.
– Creatinine clearance less than 30 mL/min.

### Pre-therapeutic work-up

Patients eligible for the trial must have performed: diagnostic upper intestinal endoscopy with biopsies, thoraco-abdominal-pelvic CT scan, laboratory exams: serum carcinoembryonic antigen (CEA), CA19.9, hemoglobin, leukocytes, neutrophils, platelets, glycemia, AST, ALT, lactate dehydrogenase (LDH), total bilirubin, alkaline phosphatase, serum albumin, total protein, plasmatic activated partial thromboplastin time (APTT), PT, creatinine clearance, and serum creatinine and pregnancy serological test.

Patients should undergo to staging laparoscopy in one of the participating centers to define the peritoneal involvement.

Of note, during explorative laparoscopy PCI according to Sugarbaker will be assessed based on peritoneal lesions size and distribution. More in detail, each location of a 13 points list (central abdominal wall, epigastrium, right lower abdominal wall, right upper abdominal wall, right flank, left lower abdominal wall, left upper abdominal wall, left flank, pelvis, upper jejunum, lower jejunum, upper ileum, and lower ileum) received a peritoneal carcinomatosis grade ranging from 0 to 3, i.e. no visible carcinomatosis, isolated tumor nodules, multiple tumor nodules, and confluent lesions.

In case of PCI ≤6 and/or positive peritoneal cytology patient the patient is considered to enter the study protocol. After that peritoneal metastasis will be confirmed by cytological/histopathological final examination and the existence of all inclusion criteria as well as the absence of exclusion criteria are verified, patient will be randomized.

### Randomization

Once the inclusion and exclusion criteria are confirmed, each patient will be randomized in a 1:1 ratio (chemotherapy alone vs. chemotherapy plus PIPAC) to blocks of 14 using a centralized randomization list, stratified by center. Randomization list will be managed by the Medical Epidemiology and Statistics Unit, Department of Diagnostics and Public Health of the University of Verona. This list will be built using the Biostatistics Unit of the University of Verona using software (www.randomizer.org).

#### Arm A (FOLFOX)

Patients randomized in the arm A will undergo to six courses of systemic chemotherapy according to FOLFOX regimen, after these six courses of chemotherapy, radiologic restaging (CT scan), as well as a second staging laparoscopy will be performed. If a progression disease will be detected, the patient will end the trial and will undergo to II line chemotherapy regimen. If a stable disease or a partial response will be documented, after a multidisciplinary discussion, patient will undergo to either further six courses of chemotherapy or to cytoreductive surgery plus hyperthermic intraperitoneal chemotherapy (HIPEC).

#### Arm B (FOLFOX + PIPAC)

**Figure 1:**
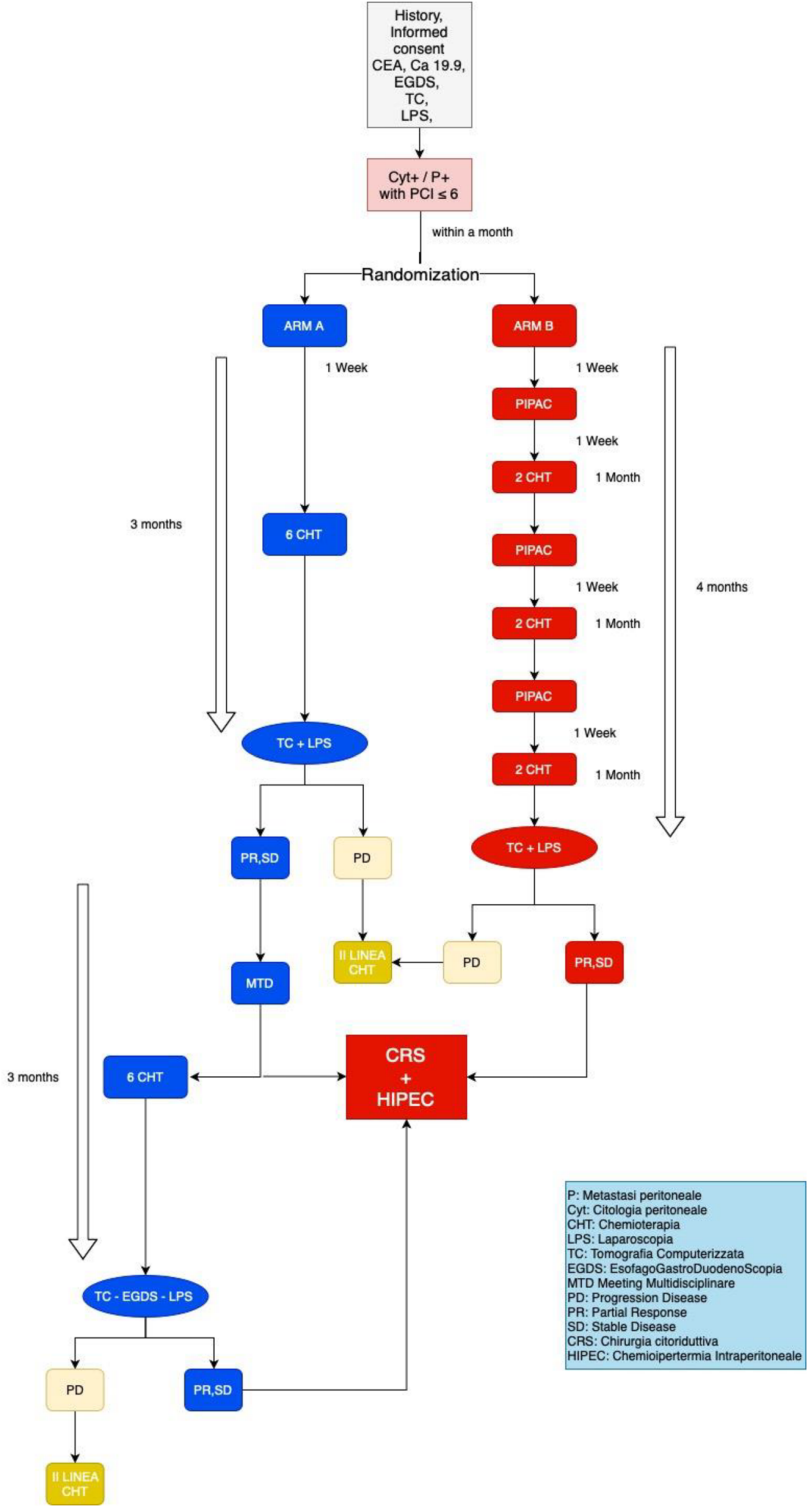
Trial flow-chart.

Patients randomized in the ARM B will undergo to 6 courses of systemic chemotherapy (FOLFOX regimen) plus PIPAC every two cycles of chemo (Figure 1). At least seven days should last between each PIPAC and the next chemotherapy course, and at least 14 days should last between the chemotherapy course and the next PIPAC. After six courses of chemotherapy and 3 PIPACs procedure, a radiologic restaging (CT scan) as well as a laparoscopic reassessment will be performed. If a progression disease will be detected, the patient will end the trial and will undergo to II line chemotherapy regimen. If a stable disease or a partial response will be documented, patient will be treated with cytoreductive surgery plus HIPEC.

For better understanding of protocol scheme, see Figure 1.

### Treatments

#### PIPAC-procedure

A minilaparotomy of 3 cm is performed, usually in the midline. Then, a 5 or a 10–12 mm balloon trocar is inserted under “finger protection” [6] usually in the right side and the fascia of the minilaparotomy, is then closed. The abdomen is insufflated with CO_2_ and tightness is controlled with saline solution in the minilaparotomy. CO_2_-bubbling documents incomplete closure. A second 10–12 mm trocar is then introduced safely under videoscope control usually in upper left side. Ascites volume is documented, and ascites is removed sending a sample for cytological examination, an accurate exploratory laparoscopy is performed, possibly placing an additional 5 or 10–12 mmHg trocar in an area not affected by adhesions or disease, the PCI is calculated. Multiple biopsies are performed in different abdominal quadrants during the first procedure and all following procedures to ascertain tumor regression grade [7]. Then, a nebulizer CAPNOPEN© is connected to an intravenous high-pressure injector and inserted into the upper left side trocar and fixed with a 45° angle to the underlying peritoneum to allow a better spatial drug distribution pattern and a greater spraying distance between the nozzle head and the underlying small bowel peritoneum compared to the that obtained with a perpendicular nozzle position [8].

The liquid chemotherapeutic drugs (cisplatin 10.5 mg/m^2^ body surface in a total of 150 mL NaCl 0.9%; doxorubicin 2.1 mg/m^2^ body surface in a total of 50 mL NaCl 0.9%) are then injected through remote control with a flow rate of 0.7 mL/sec with a maximum operating pressure of 200 psi (13 bar) into the constant capnoperitoneum of 12 mmHg. After an aerosol exposure phase of 30 min, the aerosol is evacuated via a closed aerosol waste system. Finally, trocars are retracted, and laparoscopy ended. No drainage of the abdomen is applied.

#### Intravenous chemotherapy

FOLFOX regimen systemic chemotherapy will be administered to each patient in both arms.

Arm A will receive only systemic chemotherapy according to this scheme: Oxaliplatin 85 mg/m^2^, dL, over 2 h, leucovorin* 400 mg/m^2^, d1, i.v. over 2 h, 5-FU 400 mg/m^2^ in bolus and 5-FU 2.400 mg/m^2^, d1, i.v. over 46 h.

Arm B will be treated with systemic chemotherapy plus PIPAC procedure according to this scheme (Figure 1).

PIPAC with cisplatin 10.5 mg/m^2^ and doxorubicin 2.1 mg/m^2^; chemotherapy with the same way of arm A.

At least seven days should last between each PIPAC and the next chemotherapy cycle, and at least 14 days should last between the chemotherapy cycle and the following PIPAC procedure.

#### Laparoscopy for final evaluation

Restaging laparoscopy is performed at 3 months e.g. 1 month after the third PIPAC application for the experimental group. PCI and ascites volume are evaluated, tumor biopsy is taken to ascertain tumor regression. In case of response or stable disease after the treatment, the experimental group is candidate to an explorative laparotomy: if the response and the possibility of local radical resection (duodenal involvement, pancreatic involvement, and infiltration of hepatoduodenal ligament) is confirmed, the patient is candidate to gastrectomy and cytoreductive surgery that mean radical-intent resection of all the volume and sites of disease that were present at time of first diagnosis. If a R0 is reached, then HIPEC is added. Conversely, in the standard group the decision on whether to submit the patient to radical-intent surgery or continue with chemotherapy is discussed by the multidisciplinary team after at least 3 courses of systemic chemotherapy, as this is the strategy that is currently adopted in clinical practice.

## Results

### Primary outcome

Secondary resectability rate (%) evaluated as the rate of patients of the two arms that get radical intent surgery (cytoreductive surgery and HIPEC). The resectability is assessed by surgical exploration in all patients who have achieved stable disease or a response after treatment in which a macroscopic R0 resection can be potentially obtained.

### Secondary outcomes

– Overall survival (OS).
– Progression-free survival (PFS).
– Disease-related survival (DRS).
– Histological response (peritoneal regression grade score and tumor regression grading (TRG) sec. Mandard and Becker).
– Quality of life (QoL).
– Adverse events.
– Incremental cost-effectiveness ratios (ICER).

### Sample size calculation

According to the current literature, considering a resectability rate of 50%^9^ for patients undergoing chemotherapy alone (arm A) and 80% in the experimental arm (arm B), we need 88 patients (44 per group) to achieve 80% potency by performing an exact bidirectional Fisher test with an alpha of 5%.

Expecting a dropout rate of 10% it will be necessary to recruit 98 patients, 49 for each arm. A very similar recruitment capacity is envisaged in the different centers.

### Study duration

#### Recruitment

The enrollment of patients will last about three and a half years from the date of approval. An enrollment of about 30 patients per year is expected in the seven centers involved. If the enrollment is lower than expected, up to ten centers will be recruited and the enrollment period will be extended. These latter changes will be the subject of any future substantial amendment to the current study protocol.

#### Patient involvement

Patients will be involved in the study from the time of enrollment, the duration of treatment (from a minimum of 3 months to a maximum of 6 months) and for the next 3 years of follow-up.

#### Total duration

The clinical trial will last a total of six and a half years.

#### End of clinical trial

The end of data collection coincides with the achievement of a 3-year follow-up for the last patient enrolled. An additional year will be needed for the analysis of the data and the publication of the results.

## Discussion

Peritoneal metastasis from gastric cancer is an emergent problem. During the last decades, in Western countries, tumors located in the distal third of the stomach are decreasing in favor of locally advanced proximal and diffuse-type tumors with a higher risk of peritoneal dissemination [9]. Moreover, there is evidence of an increasing incidence of stomach cancer among young adults (aged <500 years), especially of diffuse/poorly cohesive subtypes with a high affinity for peritoneum [9].

Accordingly, in clinical practice, young patients with peritoneal metastases are increasing and the management of these patients is currently a great challenge for surgical oncologists. Nowadays, the standard of treatment for gastric cancer with peritoneal metastasis is the systemic chemotherapy with a median overall survival of 8–13 months [1, 10], [11], [12]. There is increasing evidence that some patients with stage IV GC but low metastatic burden, could benefit from a combined, aggressive, treatment including systemic chemotherapy followed by radical surgery [13]. These cases are defined as “oligomestatic” GC. In addition to surgery, local chemotherapy into the peritoneum seems to be the best choice in oligometastatic gastric cancer patients with peritoneal involvement. The CYTO-CHIP trial [14] showed that in selected patients with peritoneal metastases from GC, the HIPEC after a radical surgery offers better outcomes than surgery alone, both in terms of survival and recurrence rate. Moreover, that study showed a better survival and a lower recurrence rate after combined therapy with HIPEC both in patients with poorly cohesive gastric carcinoma group (PCC group) and the non-PCC group. However, the benefits were more evident for patients with non-poorly cohesive gastric carcinoma or absence of a signet ring cell component (non-PCC group) which had better overall survival than PCC group. In patients with non-PCC, median OS was 34.5 months with CRS-HIPEC and 14.3 months without HIPEC (p=0.008). OS rates at 1, 3, and 5 years were 76.3, 48.3, and 38.6 percent, respectively, in the CRS-HIPEC group vs. 54.5, 22.0, and 18.4 per cent in the CRS-only group.

Moreover, in the CYTO-CHIP trial [14] the survival benefit was also related to the peritoneal cancer index status of patient. Patients with PCI ≤6 for PCC group and with PCI ≤12 for non-PCC group had the best benefits from HIPEC after radical surgery. Based on the results of this trial, the best treatment for selected patients with low burden peritoneal disease currently consists in systemic chemotherapy followed by radical surgery and HIPEC. Nevertheless, as indirectly suggested by the subgroup analyses of the CYTO-CHIP trial, poorly cohesive gastric carcinoma needs even more aggressive treatments, especially at intraperitoneal side.

In this setting, a new intraperitoneal chemotherapy delivery technique, PIPAC, born in 2011 could have a role. It consists in applying a cytotoxic solution (cisplatin and doxorubicin) nebulized with a micropump into the abdominal cavity for 30 min, through laparoscopic access and a normothermic capnoperitoneum with a pressure of 12 mmHg. It was shown to significantly improve the intraperitoneal drugs delivery, patient’s outcome, and survival with preserved QoL in palliative settings. Indeed, PIPAC has been used for PM of various origins, with encouraging results in gastric cancer [15], [16], [17]. The low toxicity and the low surgical impact of this new procedure, allows using it in combination with systemic chemotherapy in a bidirectional setting without increasing the drug toxicity [3, 16, 18]. Based on above, we designed the present trial focusing on peritoneal-only oligomestastatic GC patients that are those who would potentially benefit more from “intensive” bidirectional chemotherapy treatment both from systemic and intraperitoneal sides. In the experimental arm of this trial, three PIPAC procedures (1 PIPAC every 2 courses of standard chemotherapy), have been added to standard systemic chemo. Moreover, patients in the experimental arm will undergo to radical-intent surgery unless progression disease is detected, while in the standard treatment arm, radical-intent surgery will be possible on the bases of current practice, after multidisciplinary tumor board discussion. In summary, we aim to compare two strategies: the current practice and a novel therapeutic path for oligometastatic peritoneum-only gastric cancer patients where the implementation of local disease control given by intraperitoneal chemotherapy, would hopefully improve the resectability rate and the OS, PFS DRS as well as quality of life of patients.
